# Strictures on the Destruction of the Nerves in the Treatment of Carious Teeth

**Published:** 1849-07

**Authors:** 


### Collectanea.

*Strictures on the Destruction of the Nerves in the Treatment of Carious Teeth.*—Dr. Mitchell, in a communication to the London Lancet, after remarking that "there is no department of practice in which so many pernicious errors are continually permitted to go uncorrected, as that of dental surgery," refers to a report of a case published in the Lancet of Nov. 24th last, in which the writer states—"I then extracted two superior molares, and cut across and destroyed the nerves of the four upper incisores."

"I am perfectly aware," says Dr. Mitchell, "that the latter of these operations is of every-day occurrence among dentists, but it is, nevertheless, one that cannot, for a moment, be defended on scientific grounds."

In the case detailed, the four upper incisores were, doubtless, affected with caries. Now, the natural progress of dental caries is from decay of the crown to destruction of the pulp, and gradual wasting of the fang; the latter process being uniformly accompanied with more or less of morbid action in the periosteum of the socket. The most painful of these several stages is, beyond doubt, that in which the pulp is affected, but the subsequent stage—involving, as it does, the periosteum—is infinitely more pernicious to the health and well-being of the rest of the mouth. A single quotation from Sir Benjamin Brodie must set this at rest. "The inflammation on which the tooth-ache depends," says that gentleman, in one of his clinical lectures, "terminates, as it always does, in the death of the pulp of the tooth. Then the whole tooth dies, and is now like a portion of dead bone, or any foreign substance, stuck in the jaw."—(*Medical Gazette*, vol. xv, p. 347.) Operations, therefore, for the artificial destruction of the pulp of a carious tooth, have no higher object in view than the hurrying of the disease through its most painful stage, in order the sooner to induce the more advanced and far more injurious one. They are, in fact, operations for the more speedy production of stumps; their sole aim is, to remove pain at the expense of a greater evil. As such, it is evident that they have nothing in common with the operations of general surgery, which have for their object ultimate benefit, though purchased by immediate pain; and it is but natural to suppose that they owe their origin to the urgency of the patient rather than the suggestions of science.

"When a tooth is so far gone as to be incapable of restoration without destroying the pulp, the sooner it is extracted the better, for the mouth is sure to suffer ultimately in proportion to the number of dead teeth allowed to remain."

[We are inclined to think Dr. Mitchell cannot have judged the practical part of his profession to any great extent—if so, he would have known that by covering the pulp of the tooth with a concave piece of gold, the size of the cavity, and then plugging the cavity with gold, a tooth may be rendered serviceable for many years; and without being liable to the supposed horrors detailed even by a Sir B. Brodie or Dr. Mitchell ]

